# Study on the Effect of Koumiss Extract in Alleviating Non‐Alcoholic Fatty Liver Disease in Zebrafish Model by Improving Mitochondrial Function and Inhibiting Fat Deposition

**DOI:** 10.1002/fsn3.71582

**Published:** 2026-02-24

**Authors:** Sachula Baoyin, Qinglan Bao, Xiong Ling, Biligetu Wang, Xiaohong Bai, Meng Meng, Yingsong Chen, Tegexibaiyin Wang

**Affiliations:** ^1^ Department of Wuliao Rehabilitation Inner Mongolia International Mongolian Hospital Hohhot China; ^2^ Mongolian Medicine College Inner Mongolia Medical University Hohhot China; ^3^ Engineering of the Ministry of Education of Mongolian Medicine Inner Mongolia Minzu University Tongliao China; ^4^ Mongolian Medicine Functional Food Research and Development Center Laboratory Affliated Hospital of Inner Mongolia Minzu University Tongliao China

**Keywords:** HepG2, koumiss, mitochondrial complex I, NAFLD, zebrafish model

## Abstract

Non‐alcoholic fatty liver disease (NAFLD) has emerged as a significant health issue due to the pathological accumulation of fat in the liver in the absence of excessive alcohol intake, with mitochondrial dysfunction being a critical underlying mechanism. This study aimed to evaluate the therapeutic potential of koumiss extract, along with 2‐furanic acid and α, α‐trehalose, in modulating mitochondrial function and mitigating fat deposition in NAFLD. Utilizing molecular docking techniques, we assessed the binding affinities of these compounds to mitochondrial complex I assembly (MCIA) proteins, while establishing both in vitro (HepG2 cell line) and in vivo (zebrafish model) NAFLD models to measure lipid accumulation and related biochemical parameters, including triglyceride (TG), total cholesterol (TC), and lactate dehydrogenase (LDH) levels, alongside the expression profiles of MCIA proteins. Our results demonstrated that koumiss extract, 2‐furanic acid, and α, α‐trehalose significantly decreased TG and LDH levels indicative of steatosis in HepG2 cells, while also reducing the expression of MCIA‐related proteins. In vivo experiments using a zebrafish NAFLD model demonstrated pronounced liver steatosis in the model group. Treatment with koumiss extract, 2‐furanic acid, and α, α‐trehalose significantly alleviated liver steatosis and reduced TG and TC levels. Furthermore, mRNA expression levels of ACAD9, ECSIT, NDUFAF1, and NDUFAF2 were significantly downregulated in the treatment groups. Koumiss extract, 2‐furanic acid, and α, α‐trehalose exhibit significant effects in reducing MCIA‐related proteins and steatosis in NAFLD models. Consequently, these results suggest that koumiss extract and its analogs hold promise as therapeutic agents for NAFLD, potentially enhancing liver lipid homeostasis.

## Graphical abstract

Koumiss extract, 2‐furoic acid, and α,α‐trehalose alleviate NAFLD via modulating MCIA‐related proteins. This study illustrates the proposed mechanism by which koumiss extract and its bioactive analogs—2‐furoic acid and α,α‐trehalose—ameliorate NAFLD. Molecular docking analyses revealed strong binding affinities of these compounds to MCIA proteins. In both HepG2 cellular and zebrafish larval NAFLD models, treatment with the koumiss extract, 2‐furoic acid, and α,α‐trehalose significantly reduced lipid accumulation, TG, TC, and LDH levels. Mechanistically, the compounds downregulated mRNA expression of MCIA‐related factors including ACAD9, ECSIT, NDUFAF1, and NDUFAF2. These findings support the therapeutic potential of koumiss extract and its constituents in restoring mitochondrial function and hepatic lipid homeostasis.

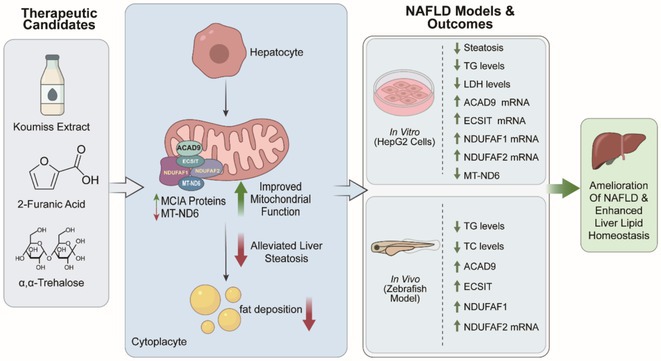

## 1 Introduction

Non‐alcoholic fatty liver disease (NAFLD) is a clinical pathological condition characterized by excessive fat accumulation in liver cells, excluding alcohol and other recognized liver injury factors. It is closely associated with insulin resistance and genetic predisposition (Tarantino 2022). The estimated global incidence of NAFLD is 47 cases per 1000 population, with an upward trend over recent years (Teng et al. 2023). The pathological mechanisms underlying NAFLD involve multiple factors, including lipid metabolism disorders, insulin resistance, inflammatory responses, and cellular apoptosis. The primary pathogenesis of NAFLD is attributed to abnormal lipid accumulation in the liver, which is strongly associated with obesity, diabetes, and high‐fat dietary habits.

Mitochondrial dysfunction is a hallmark of NAFLD and is characterized by altered liver mitochondrial morphology and impaired oxidative capacity (Zhao et al. 2023; Ramanathan et al. 2022). This dysfunction is intricately linked to disruptions in lipid metabolism and oxidative stress. The progression of NAFLD, from simple steatosis to non‐alcoholic steatohepatitis (NASH), may culminate in liver fibrosis, cirrhosis, or hepatocellular carcinoma (HCC) (Ota 2021; Tan et al. 2022). During this progression, excessive lipid accumulation triggers oxidative stress and inflammatory responses, exacerbating liver injury (Fan et al. 2023). The assembly of mitochondrial complex I (CI) is critical for oxidative phosphorylation (OXPHOS), and CI dysfunction can amplify oxidative stress, thereby aggravating lipid metabolism abnormalities in the liver (Papa et al. 2012; Vogel et al. 2007). Evidence indicates that mitochondrial dysfunction contributes to the progression of NAFLD, particularly in cases of lipid accumulation and hepatocyte damage, where mitochondrial OXPHOS capacity significantly diminishes, exacerbating the condition (Yin et al. 2019). Additionally, the regulation of lipid metabolism by mitochondria plays a pivotal role in NAFLD pathogenesis. Thus, exploring the association between MCIA, OXPHOS, and lipid metabolism is essential for elucidating the pathological mechanisms of NAFLD.

Patients with NAFLD are often asymptomatic in the early stages, with many cases identified incidentally during routine medical examinations. As the disease advances, patients may experience non‐specific symptoms such as fatigue, abdominal discomfort, and anorexia, which are frequently overlooked or misattributed to other conditions (Yamamura et al. 2021). Therefore, early diagnosis and timely intervention are critical for effective management. There are currently no Food and Drug Association‐approved drugs for treating NAFLD, emphasizing the need to explore its pathogenesis for the development of new therapeutic interventions.

Natural products have garnered considerable attention in recent years due to their potential therapeutic benefits. Koumiss is a medicinal and edible dairy product made by naturally fermenting fresh mare's milk. As early as the 14th century, the first official monograph on dietary nutrition and dietary therapy in Chinese history, “Yin Shan Zheng Yao,” (Husigui 2008) as well as the 13th‐century historical literary masterpiece, “The Secret History of The Mongols,” (Bayaer Compile 1980) both recorded the high status and long‐standing tradition of sour mare's milk in the lives of the Mongolian people. Additionally, koumiss has been shown to enhance immune function and regulate blood pressure, with positive effects on the kidneys, endocrine glands, gastrointestinal tract, liver, nervous system, and vascular system (Kocyigit et al. 2024; Adil et al. 2022; Musaev et al. 2021). Transcriptomics was employed to investigate the influence of Koumiss on both hyperlipidemic patients and individuals with good health. Meanwhile, serum pharmacological and chemical analyses were conducted on blood samples from hyperlipidemia patients, we found that koumiss contains linoleic acid and alpha‐Linolenic acid, as well as various bioactive compounds. Koumiss exhibits the potential to lower blood lipid levels and prevent the onset of hyperlipidemia. This beneficial effect may be linked to the downregulation of the ACA9 gene, which, in turn, promotes fatty acid metabolism (Bao et al. 2025) and offers diverse health benefits such as antioxidant, antimicrobial, antifungal, anti‐inflammatory, anti‐diabetic, and anti‐atherosclerotic properties (Şanlier et al. 2019). However, the precise mechanisms underlying the effects of koumiss in NAFLD remain unclear and require further investigation. Previous studies have demonstrated that koumiss exhibits lipid‐lowering effects and promotes OXPHOS in liver tissue. Nonetheless, the specific mechanisms through which koumiss alleviates steatosis in NAFLD models, both in vivo and in vitro, remain unknown.

This study aims to investigate the therapeutic potential of koumiss extract, 2‐furanic acid, and α, α‐trehalose in addressing NAFLD through the modulation of mitochondrial function. By employing molecular docking techniques, we will evaluate the binding affinities of these compounds to MCIA proteins. Additionally, we will establish both in vitro and in vivo models of NAFLD to assess lipid accumulation and related biochemical parameters, including triglyceride (TG), total cholesterol (TC), and lactate dehydrogenase (LDH) levels. The pathogenesis of NAFLD is complex, involving intricate interactions between OXPHOS and lipid metabolism. Given the limitations of current pharmacological treatments, natural products such as koumiss may represent promising alternatives. Koumiss extracts and their active components demonstrate considerable potential in regulating mitochondrial function and improving lipid metabolism. These findings offer new perspectives and potential therapeutic targets for NAFLD treatment, and future research should focus on further validating the clinical efficacy and mechanisms of these compounds.

## 2 Material and Methods

### 2.1 Koumiss and Extract Preparation

Koumiss, product code: DBS15/013‐2019, Inner Mongolia Masu Dairy Co., Ld. The method is to collect fresh mare's milk, filter to remove impurities, preheat to 50°C–60°C, sterilize at 95°C for 10 min, and ferment at a constant temperature to prepare Koumiss beverage, stored at 4°C. For the preparation of Koumiss extract, centrifuge Koumiss at 3000 rpm for 10 min. Take the supernatant and place it in a beaker, ultrasound at 37°C for 1 h. After ultrasound, add 4 times anhydrous ethanol and let it sit overnight. The next day, filter at room temperature, rotary evaporate, and then freeze‐dry.

### 2.2 Cell Culture

HepG2 cells were cultured in DMEM medium supplemented with 10% fetal bovine serum (FBS), 100 units/mL penicillin, and 100 μg/mL streptomycin in a 37°C incubator with 5% CO₂. The culture medium was replaced every 2 days. After 24 h of incubation in 96‐well plates, the old medium was discarded, and the cells were rinsed three times with PBS. To establish a non‐alcoholic fatty liver cell model, the cells were cultured in DMEM medium containing 0.25 mM oleic acid (OA) for 24 h.

### 2.3 Animal Models

The animal experiment was executed with the agreement from the Ethics Committee of the author's Hospital; all methods are reported in accordance with ARRIVE guidelines. Zebrafish embryos were obtained through spontaneous mating. After cleansing, suitable embryos were selected based on their developmental stage at 4 and 24 h post‐conception. The embryos were cultivated in ZR solution, which consisted of 3.5 g of NaCl, 0.05 g of KCl, 0.025 g of NaHCO₃, and 0.1 g of CaCl₂ dissolved in deionized water to a final volume of 1 L. The pH of the water was maintained between 6.9 and 7.2; the conductivity ranged from 480 to 510 μS/cm, and the hardness ranged from 53.7 to 71.6 mg/L CaCO₃. Since the yolk sac provided sufficient nutrition, the embryos required no additional feeding for the first 9 days post‐conception. All experimental protocols adhered to the guidelines of the Association for Assessment and Accreditation of Laboratory Animal Care (AAALAC). Zebrafish larvae were randomly distributed into 6‐well plates, with 10 larvae per well. To establish the NAFLD model, juvenile zebrafish were incubated in water containing 0.06% thioacetamide (TAA) for 72 h.

### 2.4 Main Reagents

The koumiss extract was prepared as follows: Koumiss was centrifuged at 3000 rpm for 10 min. The resulting supernatant was transferred to a beaker and subjected to ultrasonication at 37°C for 1 h. Subsequently, four times the volume of anhydrous ethanol was added, and the mixture was left to stand overnight. The following day, the mixture was centrifuged at room temperature. The supernatant was vacuum‐filtered, subjected to rotary evaporation, and then freeze‐dried.

The high‐fat, high‐sugar DMEM medium and extra‐grade FBS used for cell experiments were purchased from Gibco (Grand Island, NY, USA). Dimethyl sulfoxide (DMSO) was obtained from Beijing Solarbio Technology Co. Ltd. (Beijing, China), while oleic acid (OA) was purchased from Shanghai Sangong Biological Engineering Co. 2‐Furoic acid was sourced from Beijing Solepol Science and Technology Co. Ltd. (Beijing, China), and α, α‐Trehalose was procured from Shanghai Macklin Biochemical Technology Co. (Shanghai, China). All reagents were sterilized using 0.22 μm microporous membranes prior to use.

The RevertAid First Strand cDNA Synthesis Kit was obtained from Thermo Fisher Scientific (New York, USA). The Easy II Protein Quantitative Kit (BCA) was purchased from Beijing Chuanshijin Biotechnology Co. Ltd. (Beijing, China). Recombinant anti‐NDUFAF1 (ab79826) antibody was acquired from Abcam Antibodies (Cambridge, UK), while GAPDH XP Rabbit (5174S) and Anti‐rabbit IgG (H + L) DyLight 800 4× PEG Conjugate (5151P) secondary antibodies were procured from Cell Signaling Technology (Boston, USA). NDUFAF2 Polyclonal Antibody (PA5‐63019) and MT‐ND6 Polyclonal Antibody (PA5‐109993) were purchased from Thermo Fisher Antibody Company (New York, USA). Primer sequences were designed and sourced from Shanghai Sangong Bioengineering Co. Ltd. (Shanghai, China). The HepG2 cell line was obtained from Saibaikang Biotechnology Co. (Shanghai, China).

Thioacetamide for zebrafish experiments was purchased from Beijing Solarbio Technology Co. Ltd. (Beijing, China). AB‐strain zebrafish were supplied by the National Zebrafish Resource Center (Wuhan, China) under laboratory animal license number NM‐LL‐2023‐08‐14‐01. All other reagents were obtained from local suppliers unless otherwise specified.

### 2.5 Molecular Docking

The TCMSP platform was utilized to optimize active substances by reducing their energy and generating three‐dimensional structures while maintaining the mol2 format. The PDB database (http://www.rcsb.org/) provided the PDB format files for commonly targeted pharmacological disease proteins. Receptors were processed using Pymol software to remove solvents and water molecules. Molecular docking was performed using AutoDock, and LIGPLOT was employed to visualize and analyze the docking data. This process automatically generated a two‐dimensional protein‐ligand interaction map, indicating atomic distances and types of interactions between receptors and ligands.

### 2.6 ELISA

Well‐grown HepG2 cells were seeded into 6‐well culture plates at a density of 5 × 10^5^ cells per well and incubated in a constant‐temperature incubator for 24 h. Subsequently, drugs at various concentrations were added to each group, with four wells per group, and the cells were cultured for an additional 24 h. The procedures for determining TG and LDH levels strictly followed the instructions provided in the reagent kits.

### 2.7 Real‐Time PCR

To ensure uniform lysis, culture media were removed from the 6‐well plates, and the wells were washed twice with pre‐cooled PBS. Trizol (1 mL) was added to each well, ensuring the cell surface was fully covered, and the plates were placed on ice for 5 min. The subsequent steps were performed in strict accordance with the Trizol kit instructions. The primer sequences of the related proteins are shown in Table 1.

**TABLE 1:** Primer sequences used for real‐time PCR.

Gene	Forward primer (5′‐3′)	Reverse primer (5′‐3′)
ACAD9	AGCACAGTCATGGATACCGTTGG	GCCGAAGCAGTAGGTGTTCTCC
NDUFAF1	CCTACTGGCAGGAGGTCAAGATTC	GCGGAAGCTCATGCTGAACATC
NDUFAF2	TTGGAAAGGAAGAACCCTCAGTGG	CCATCTCGTGGCATCCAGGATC
ECSIT	TTTGCCCAAAGACTCAACAGGTG	CCAGCCACTCCTCACATACTCC
GAPDH	GCATCCTGGGCTACACTGAG	GTCAAAGGTGGAGGAGTGGG

In the animal experiment section, the zebrafish larvae from each group were anesthetized and placed in a 1.5 mL EP tube containing 1 mL of Trizol for homogenization. The specific steps are consistent with the cell experiments. The results were normalized to the GAPDH of zebrafish larvae.

### 2.8 Western Blot

The culture medium was discarded from the 6‐well plates, and the wells were washed twice with PBS. Cells were centrifuged at 500 × g for 5 min, and the supernatant was carefully discarded. A total of 1 mL of protein lysis buffer, prepared with TPED (Protease Inhibitor Cocktail, EDTA‐free; 100× = 99:1), was added to the cell pellet. The mixture was vortexed for 15 s, incubated on ice for 30 min, and vortexed every 10 min. The lysate was collected, centrifuged at 12,000 rpm for 10 min at 4°C, and the supernatant was stored at −80°C for subsequent analysis.

### 2.9 Oil Red O Staining

Fifteen zebrafish per group were collected into staining dishes, washed twice with PBS, and fixed with 4% paraformaldehyde overnight at 4°C. The following day, the zebrafish were washed twice with PBS to remove residual fixative. The staining procedure was performed according to the instructions of the Oil Red O Staining Kit. Hepatic steatosis was examined and photographed using a somatic microscope after the stained zebrafish were transferred to 70% glycerol and stored at room temperature.

### 2.10 HE Staining

At the designated time points, 15 zebrafish per group were collected and placed into 1.5 mL EP tubes. The zebrafish were washed twice with PBS and fixed in 4% paraformaldehyde at 4°C for 4 h. Subsequently, the samples underwent dehydration, embedding, and sectioning into 3–5 μm slices. Staining was performed according to the HE staining kit instructions.

### 2.11 Zebrafish Model Whole‐Fish ELISA Detection

A total of 120 zebrafish from each group were randomly selected, homogenized with normal saline, and centrifuged at 3000 rpm for 10 min at 4°C. The supernatant was collected, and the levels of TG and TC were measured using triglyceride and cholesterol test kits. Each experiment was conducted in triplicate.

### 2.12 mRNA Expression of Mitochondrial Complex I‐Related Proteins in the Zebrafish Model Detected by Real‐Time PCR

A total of 120 juvenile zebrafish were randomly selected from each group, washed twice with pre‐cooled PBS, and homogenized with 1 mL of Trizol along the well walls. The samples were left on ice for 5 min. The subsequent steps were carried out strictly according to the Trizol kit instructions. The primer sequences of related proteins are shown in Table 2.

**TABLE 2:** Specific sequences of primers used in RT‐qPCR.

Gene	Forward primer (5′‐3′)	Reverse primer (5′‐3′)
ACAD9	ATGATGGACCGCCCTGGACTC	GCCCTCAGAACTAAAGACCTTGACC
NDUFAF1	AGGAGCAAGTTTGAGCGAGATTACC	CTTCCTGACACTCTCCACACCTTTG
NDUFAF2	CGGTTTGGTGGCTGAGTCTGTTC	GACCCTGGCTGGAATGCGTTG
ECSIT	AACCCTTACCCCGTCCCACATG	TTAGCATCCAGATCAGCAGCAATCC
GAPDH	CGCTGGTGCTGGTATTGCTCTC	GCCATCAGGTCACATACACGGTTG

### 2.13 Statistical Analysis

SPSS 24.0 software was used for analysis and processing, and the data were expressed by mean + standard deviation (±SD). Single‐factor variance (one‐way analysis of variance, ANOVA) was used. *p* < 0.05 showed that the difference was statistically significant.

## 3 Results

### 3.1 Molecular Docking Analysis of Active Ingredients With Protein Targets

**FIGURE 1:**
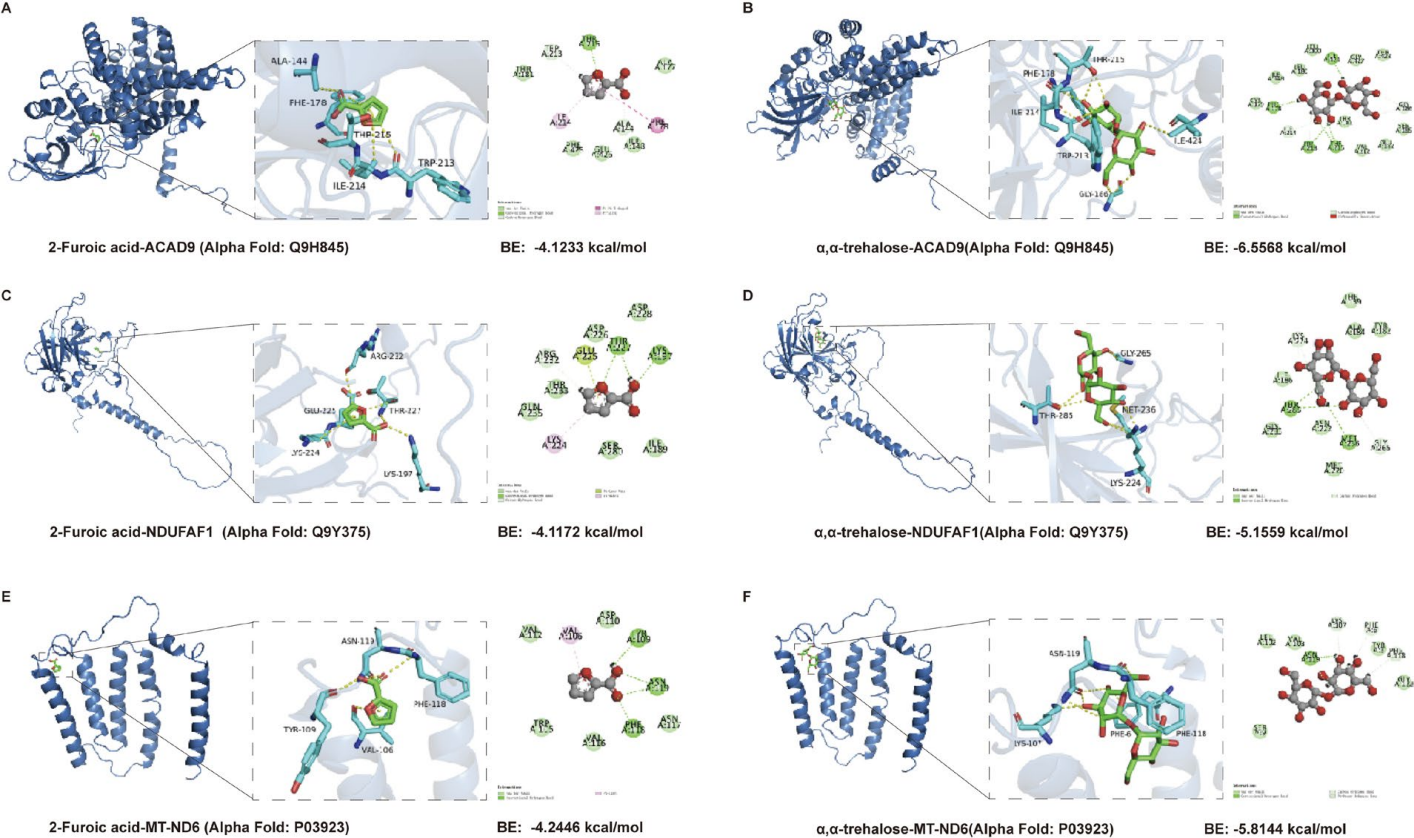
Molecular docking analysis of the bonding patterns of 2‐furoic acid and α, α‐trehalose dihydrate with key molecular targets: ACAD9, NDUFAF1, and MT‐ND6 protein. The specific docking results are as follows: 2‐furoic acid_ACAD9 protein (A), α, α‐trehalose dihydrate_ACAD9 protein (B), 2‐furoic acid_NDUFAF1 protein (C), α, α‐trehalose dihydrate_ NDUFAF1 protein (D), 2‐furoic acid_MT‐ND6 protein (E) and α, α‐trehalose dihydrate_ MT‐ND6 protein (F).

To investigate protein‐ligand interactions, the active molecule α, α‐trehalose dihydrate was subjected to molecular docking with potential targets. The results revealed significant binding interactions, with the α, α‐trehalose dihydrate_ACAD9 protein exhibiting a binding energy of −6.5568 kcal/mol. The residues of Ile424, Phe178, Trp213, Thr215, and Gly186, Trp213, Ile214 of the receptor of ACAD9 protein form hydrogen bonds with the α, α‐trehalose, and the residues of Ile424, Phe178, Trp213, Thr215, and Gly186 of the receptor of ACAD9 protein form hydrogen bonds with the α, α‐trehalose. Similarly, α, α‐trehalose dihydrate_NDUFAF1 protein displayed strong binding interactions, with a binding energy of −5.1559 kcal/mol. Thr285 and Met236 of NDUFAF1 receptor form hydrogen bonds with α, α‐trehalose, while Lys224 and Gly265 of the protein interact with α, α‐trehalose via carbon‐hydrogen bonds. The docking complexes were visualized using Pymol software, which revealed stable complexes with robust binding patterns (Figure 1).

### 3.2 Effects of Oil Red O on HepG2 Cell Model Viability and Steatosis

**FIGURE 2:**
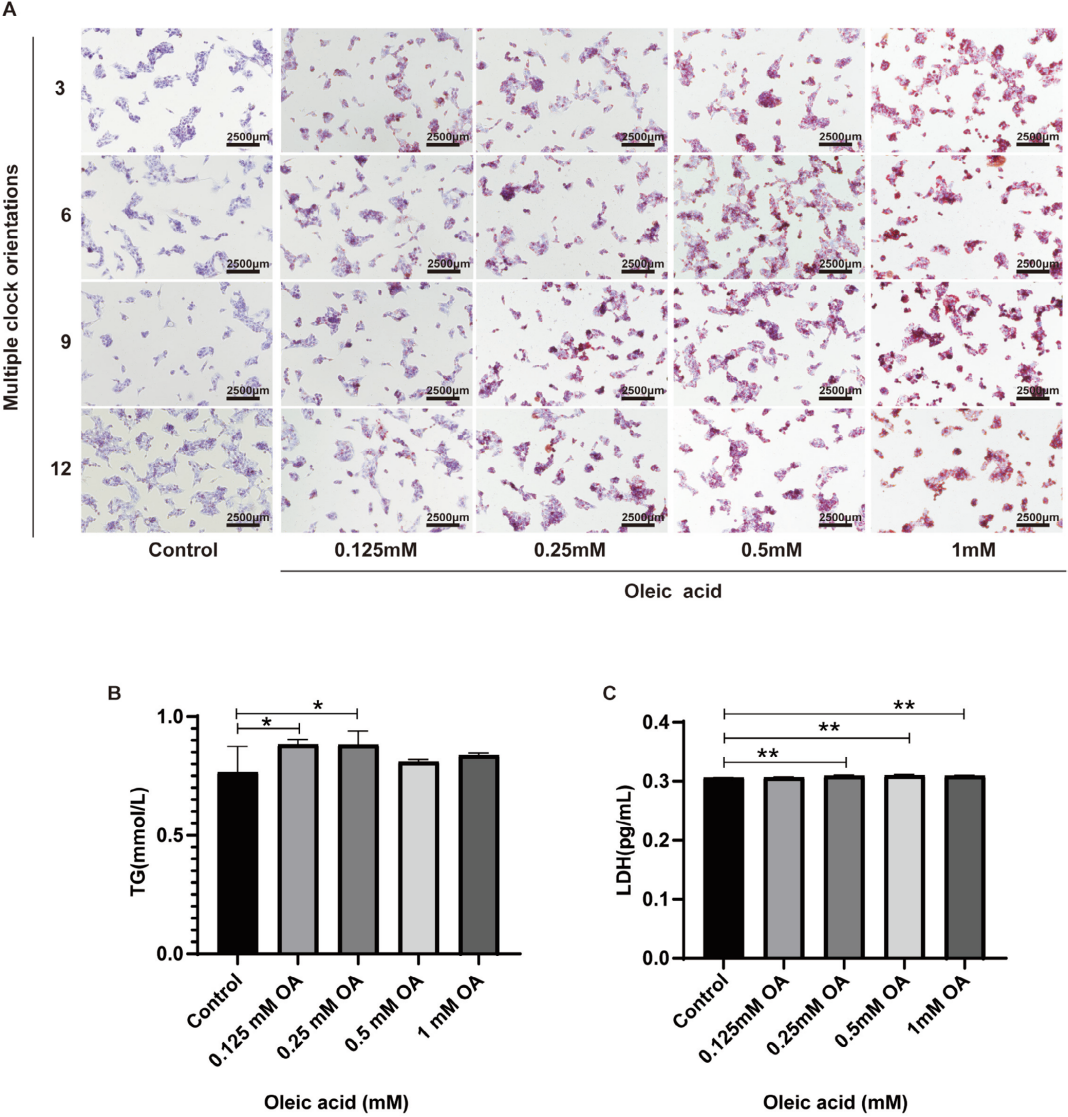
Lipid droplet formation was detected using an inverted microscope for cell culture at 40× magnification. Scale bar = 2500 μm. (A), and contents of TG (B) and LDH (C) in cell supernatant. Control group: DMEM medium; Oleic acid: DMEM medium containing different concentrations of OA (0.125, 0.25, 0.5, and 1 mM) was treated for 24 h. Multiple clock orientations: Oil Red O staining images of multiple time points (3, 6, 9, and 12 o'clock directions). Compared with the control group, *p < 0.05, **p < 0.01.

HepG2 cells were treated with varying concentrations of oleic acid (OA; 0.125–1 mM) for 24 h, after which cell viability and morphology were assessed, and the cytotoxicity of OA was evaluated. The results indicated that low concentrations of OA (0.125–0.25 mM) did not significantly affect cell viability (Figure 2A). However, at a concentration of 0.5 mM, cell viability decreased to 63%, suggesting that higher concentrations of OA could be detrimental to HepG2 cells. Oil Red O staining demonstrated that lipid accumulation in HepG2 cells was concentration‐dependent following OA treatment. The effects of various concentrations of oleic acid on TG (Figure 2B) and LDH (Figure 2C) levels in HepG2 cell supernatant are consistent with the results of Oil Red O staining. Based on these findings, a concentration of 0.25 mM OA was selected to establish the NAFLD cell model.

### 3.3 Effects of Koumiss Extract, 2‐Furoic Acid, and α, α‐Trehalose on HepG2 Cell Model Steatosis

**FIGURE 3:**
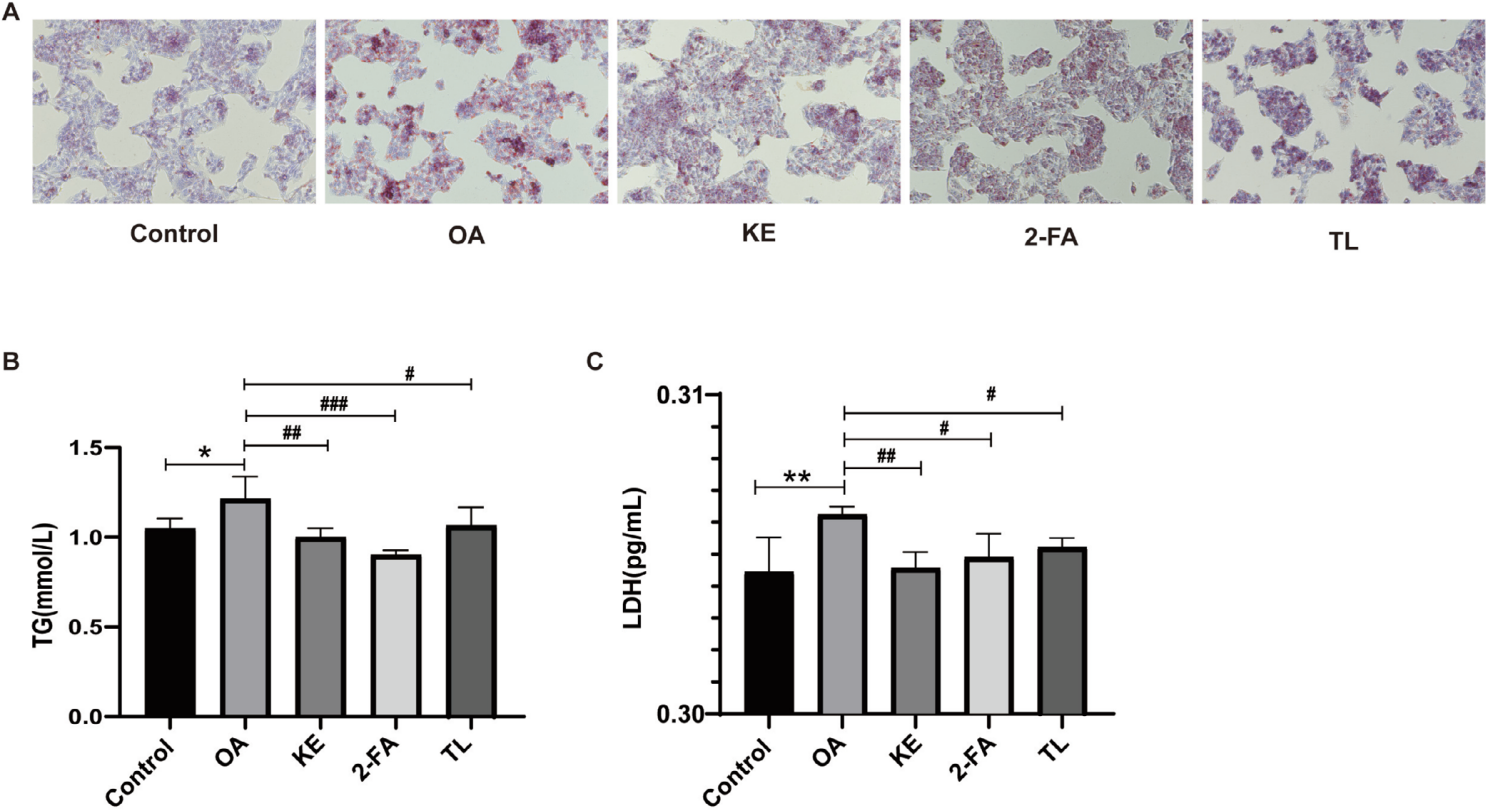
Effects of koumiss extract, 2‐furoic acid, and α, α‐trehalose on Oil Red O staining in the HepG2 cell model with steatosis (A). Effects of koumiss extract, 2‐furoic acid, and α, α‐trehalose on TG (B) and LDH (C) levels in the cell model's supernatant. *KE: Koumiss extract; 2‐FA: 2‐furoic acid; TL: α, α‐trehalose. Compared with the control group, *p < 0.05, **p < 0.01, Compared with the OA group, #*p* < 0.05, ##*p* < 0.01, ###*p* < 0.001.

Oil Red O staining results demonstrated that koumiss extract, 2‐furoic acid, and α, α‐trehalose mitigated steatosis in the HepG2 cell model to a certain extent (Figure 3A). ELISA analysis revealed that the TG and LDH levels in the model group were elevated compared with the normal group. After intervention with koumiss extract, 2‐furoic acid, and α, α‐trehalose, the TG (Figure 3B) and LDH (Figure 3C) levels were significantly reduced (*p* < 0.05).

### 3.4 Effects of Koumiss Extract, 2‐Furoic Acid, and α, α‐Trehalose on the MCIA ‐Related Proteins and MT‐ND6 Levels in HepG2 Cell Model

**FIGURE 4:**
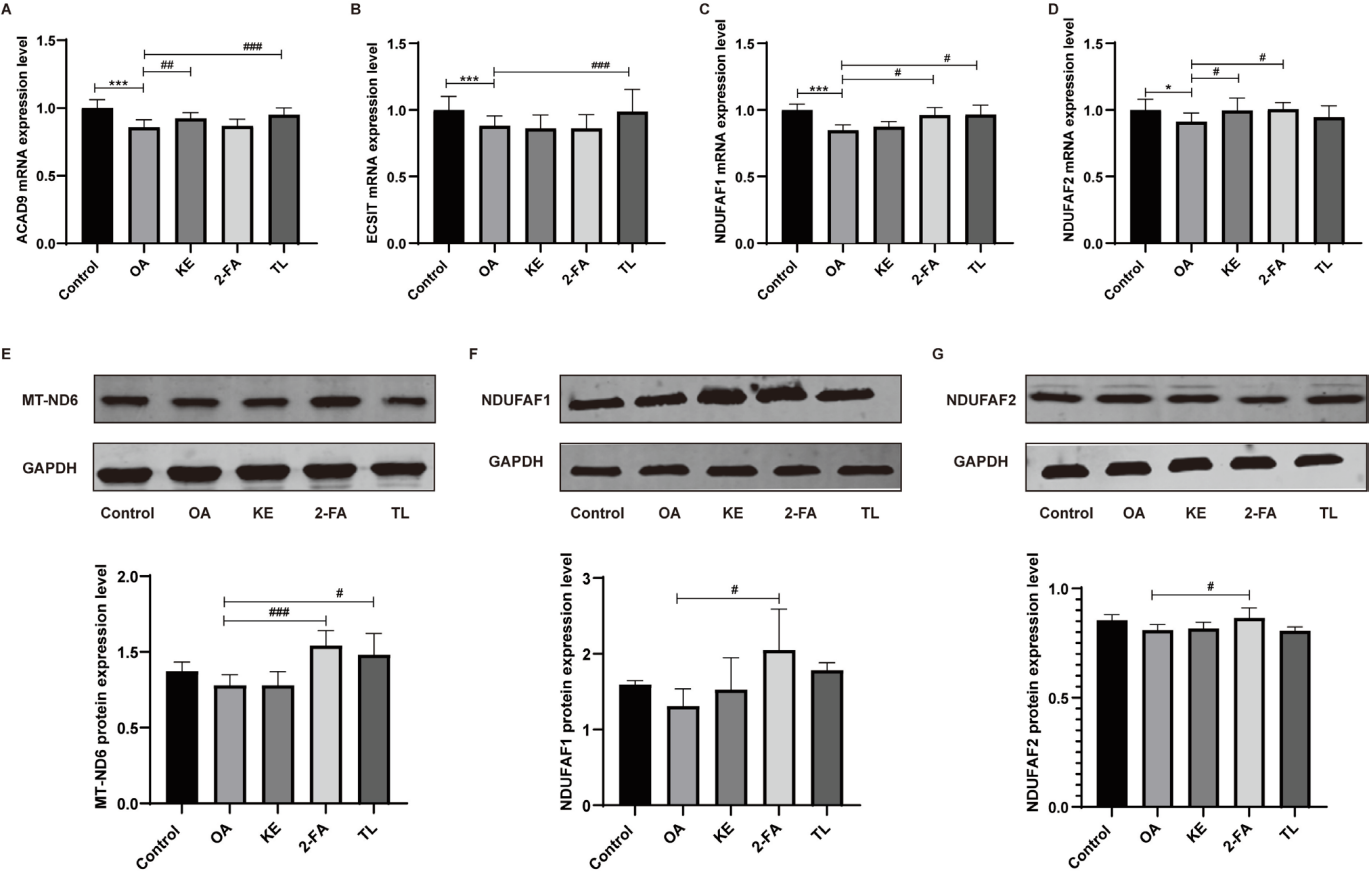
Impact of koumiss extract, 2‐furoic acid, and α, α‐trehalose on ACAD9 mRNA (A), ECSIT mRNA (B), NDUFAF1 mRNA (C), and NDUFAF2 mRNA (D) expression in the cell model. Effects of koumiss extract, 2‐furoic acid, and α, α‐trehalose on MT‐ND6 (E), NDUFAF1 (F), and NDUFAF2 (G) protein expression in the cell model (E). *KE: Koumiss extract; 2‐FA: 2‐furoic acid; TL: α, α‐trehalose. Compared with the control group, ***p < 0.001, Compared with the OA group, #*p* < 0.05, ##*p* < 0.01, ###*p* < 0.001.

Real‐time PCR analysis showed that the mRNA expression levels of ACAD9, ECSIT, NDUFAF1, and NDUFAF2 in the model group were significantly downregulated compared with the normal group. Conversely, the groups treated with koumiss extract, 2‐furoic acid, and α, α‐trehalose demonstrated marked recovery effects compared with the model group (Figure 4A–D). Western Blot results revealed that MT‐ND6 protein expression in the model group was upregulated compared with the normal group. Treatment with koumiss extract, 2‐furoic acid, and α, α‐trehalose resulted in partial recovery compared with the model group. The expression levels of NDUFAF1 and NDUFAF2 proteins were downregulated in the model group compared with the normal group. Treatment with koumiss extract, 2‐furoic acid, and α, α‐trehalose exhibited partial restoration compared with the model group (Figure 4E–G).

### 3.5 Establishment of an NAFLD Model in Juvenile Zebrafish

**FIGURE 5:**
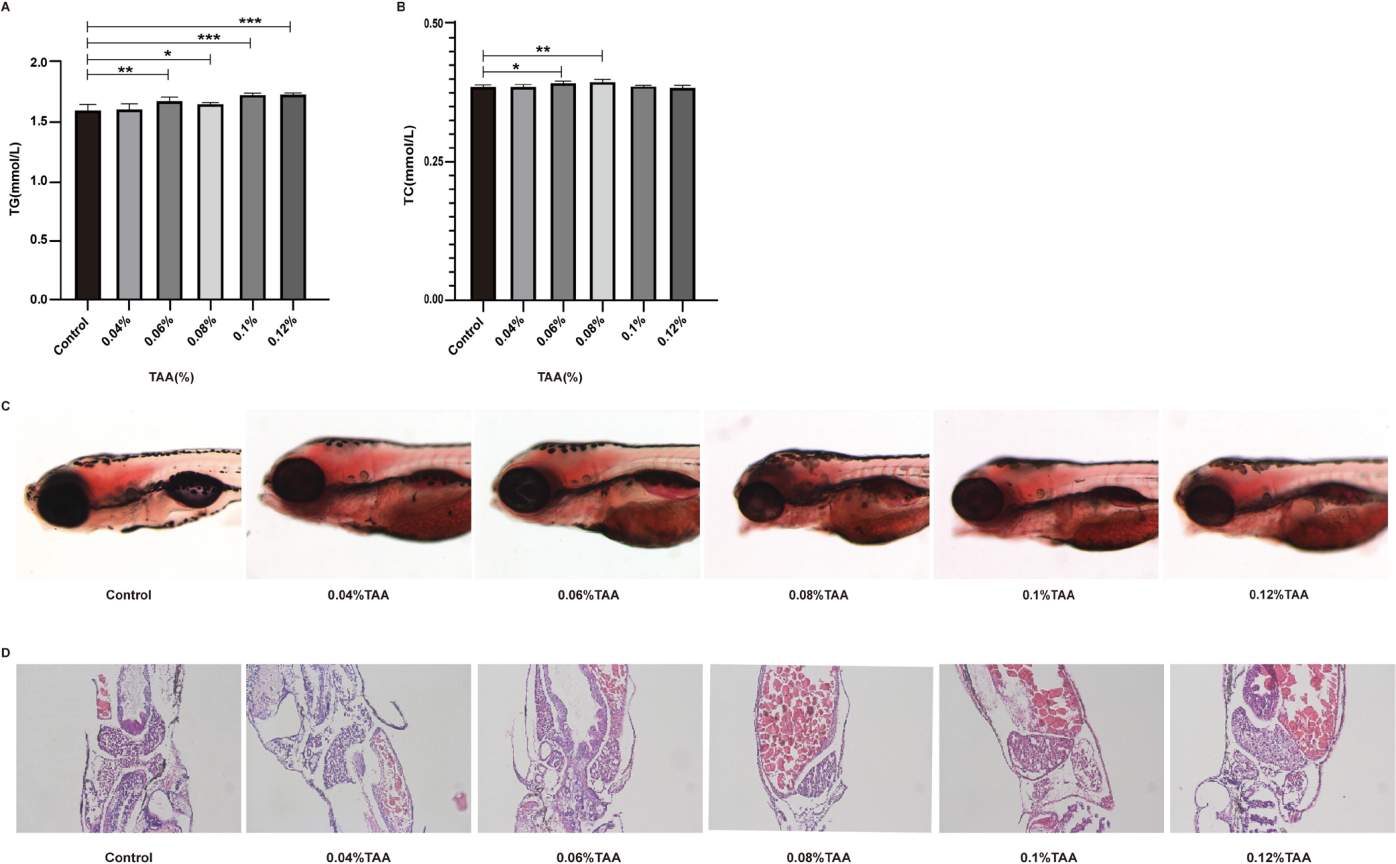
Effects of various concentrations of TAA on TG (A) and TC (B) levels in juvenile zebrafish. Effects of various concentrations of TAA on juvenile zebrafish liver staining (C) and H&E staining of steatosis (D). Each column represents the mean + SD (*n* = 3). Thioacetamide (TAA) is an inducer for establishing a juvenile zebrafish model of NAFLD. TAA: Juvenile zebrafish were exposed to different concentrations of thioacetamide (TAA; 0.04%, 0.06%, 0.08%, 0.1%, and 0.12%). Compared with the control group, *p < 0.05, **p < 0.01, ***p < 0.001.

Juvenile zebrafish were exposed to different concentrations of thioacetamide (TAA; 0.04%, 0.06%, 0.08%, 0.1%, and 0.12%) for model establishment. ELISA results for TG (Figure 5A) and TC (Figure 5B) levels indicated that the IC50 of TAA was approximately 0.06%. Oil Red O staining showed lighter staining in the control group, while staining intensity increased in a concentration‐dependent manner starting at 0.04% TAA. Compared with the control group, liver staining was more pronounced in the model group (Figure 5C). Hematoxylin and eosin (HE) staining revealed a significant increase in vacuolized hepatocytes in the model group compared with the control group. While staining intensity increased in a concentration‐dependent manner starting at 0.04% TAA. Compared with the control group, staining was more pronounced in the model group (Figure 5D). These results suggest that a TAA concentration of 0.06% is optimal for establishing the NAFLD model in juvenile zebrafish.

### 3.6 Effects of Koumiss Extract, 2‐Furoic Acid, and α, α‐Trehalose on Steatosis in Zebrafish Model

**FIGURE 6:**
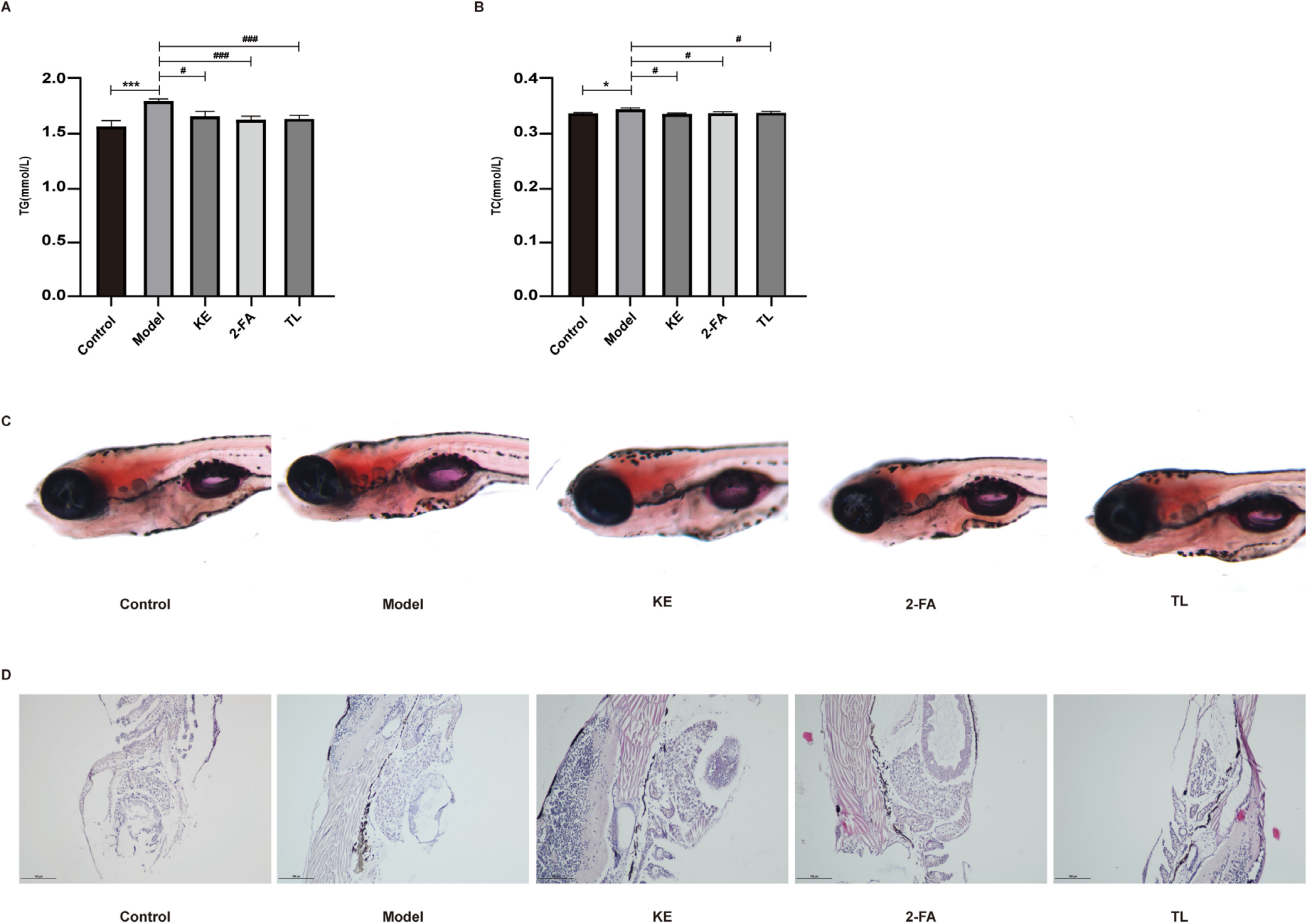
Effects of koumiss extract, 2‐furoic acid, and α, α‐trehalose on TG (A) and TC (B) levels of zebrafish model. Oil Red O staining impact on koumiss extract, 2‐furoic acid, and α, α‐trehalose on steatosis in the zebrafish model (C). H&E staining to evaluate the effect of koumiss extract, 2‐furoic acid, and α, α‐trehalose on steatosis in the zebrafish model (D). Control: 1 × ZR solution; Model: 0.06% TAA; Koumiss extract: 0.0625 g/mL; 2‐furoic acid: 80 mM; α, α‐trehalose: 80 mM. *KE: Koumiss extract; 2‐FA: 2‐furoic acid; TL: α, α‐trehalose. Compared with the control group, *p < 0.05, ***p < 0.001. Compared with the Model group, #*p* < 0.05, ###*p* < 0.001.

ELISA results indicated that TG and TC levels in the model group were significantly elevated compared with the control group. Treatment with koumiss extract, 2‐furoic acid, and α, α‐trehalose significantly reduced TG and TC levels compared with the model group (Figure 6A,B). Oil Red O staining results revealed darker liver staining in the model group compared with the control group. The liver tissue of zebrafish treated with koumiss extract, 2‐furoic acid, and α, α‐trehalose exhibited lighter staining compared with the model group, with the koumiss extract group showing the most significant improvement (Figure 6C). HE staining results showed that liver cells in the model group exhibited a greater number of vacuoles compared with the control group. Treatment with koumiss extract, 2‐furoic acid, and α, α‐trehalose markedly reduced the number of vacuolized hepatocytes compared with the model group. Among the treatment groups, the 2‐furoic acid group exhibited the most pronounced improvement (Figure 6D).

### 3.7 Effects of Koumiss Extract, 2‐Furoic Acid, and α, α‐Trehalose on the MCIA ‐Related Proteins Levels in Zebrafish Model

**FIGURE 7:**
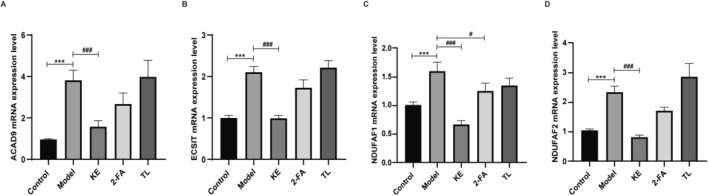
Effects of koumiss extract, 2‐furoic acid, and α, α‐trehalose on ACAD9 (A), ECSIT (B), NDUFAF1 (C), and NDUFAF2 (D) mRNA expression in the zebrafish model. Control: 1 × ZR solution; Model: 0.06% TAA; Koumiss extract: 0.0625 g/mL. KE: Koumiss extract; 2‐FA: 2‐furoic acid; TL: α, α‐trehalose. Compared with the control group, ***p < 0.001. Compared with the Model group, #*p* < 0.05, ###*p* < 0.001.

The results obtained through Real‐Time PCR revealed the Model group a significant upregulation in the mRNA expression levels of ACAD9, ECSIT, NDUFAF1, and NDUFAF2 in comparison to the Control group. Furthermore, the koumiss extract, 2‐furoic acid, and α, α‐trehalose interventions demonstrated considerable improvement when compared with the Model group (Figure 7).

## 4 Discussion

NAFLD is an increasingly prevalent liver condition characterized by excessive lipid accumulation in hepatocytes and is closely associated with obesity, diabetes, and metabolic syndrome (Rafaa et al. 2025). The pathogenesis of NAFLD is multifactorial, involving mechanisms such as insulin resistance, oxidative stress, and lipid metabolism dysregulation (Zhong et al. 2024). With the growing global prevalence of obesity, NAFLD has emerged as a significant public health concern related to chronic liver diseases (Trandafir et al. 2020). Dysregulation of hepatic lipid metabolism is a pivotal mechanism in the onset of NAFLD. Impaired fatty acid metabolism in the liver contributes to the excessive accumulation of lipids, triggering steatosis and hepatocyte damage (Barbier‐Torres et al. 2020; Huh and Saltiel 2021). Hepatic steatosis not only disrupts normal liver function but also induces inflammatory responses, which may lead to hepatocellular injury and fibrosis (Haga et al. 2019). For instance, dietary intake of excessive fats and sugars significantly exacerbates hepatic fat deposition, increasing the risk of NAFLD (Weiner et al. 2023). Thus, targeting hepatic lipid metabolism presents a promising approach for NAFLD treatment. The mitochondrial respiratory chain, particularly complexes I and III, also known as NADH–ubiquinone oxidoreductase, is implicated as a critical factor in NAFLD pathogenesis. MCIA activity represents a rate‐limiting step in the mitochondrial respiratory chain, playing a vital role in regulating oxidative phosphorylation (OXPHOS). ACAD9 is a multifunctional enzyme that contributes significantly to fatty acid β‐oxidation and MCIA. As a dehydrogenase, ACAD9 facilitates the dehydrogenation of acyl‐CoA to 2,3‐enoyl‐CoA, a key step in energy metabolism. Additionally, ACAD9 functions as a chaperone protein in CI assembly, interacting with multiple assembly factors to ensure proper complex formation (Xia et al. 2021). Studies indicate that ACAD9 forms a complex with assembly factors such as ECSIT and NDUFAF1, playing a central role in CI biosynthesis (Formosa et al. 2020). Deficiency in ACAD9 disrupts CI assembly, impairing mitochondrial energy production and overall cellular metabolic function (Xia et al. 2021). Moreover, the absence of ACAD9 compromises the structural and functional integrity of the mitochondrial membrane, adversely affecting cellular energy homeostasis and survival (Li et al. 2025). Hence, ACAD9 is a crucial regulator of mitochondrial functionality.

Koumiss, widely consumed in nomadic cultures, contains bioactive components like 2‐furoic acid and α, α‐trehalose. This beverage is renowned for its immune‐enhancing properties and benefits to cardiovascular, digestive, and endocrine systems (Afzaal et al. 2021). This study aimed to investigate the therapeutic potential of koumiss extract and its active components—2‐furoic acid and α, α‐trehalose—on NAFLD, with a specific focus on their regulatory effects on MCIA proteins. The results showed that molecular docking indicated that 2‐furoic acid and α, α‐trehalose could stably bind to ACAD9, NDUFAF1, and MT‐ND6 proteins, suggesting their potential to improve NAFLD by directly regulating the function of these target proteins. Researchers utilized OA‐induced HepG2 cells and TAA‐induced NAFLD zebrafish larvae to investigate the effects of koumiss extract, 2‐furoic acid, and α, α‐trehalose on steatosis. Post OA or TAA induction, HepG2 cells and liver cells of zebrafish larvae exhibited extensive lipid droplets, which were stained orange with Oil Red O. Treatment with koumiss extract, 2‐furoic acid, and α, α‐trehalose reduced intracellular fat formation (Figures 3A and 6D). Post‐TAA induction, zebrafish larvae showed numerous bubble‐like lesions in liver cells, which were diminished following these treatments (Figure 6D). Compared to the control group, OA‐induced cell models demonstrated increased levels of TG (Figure 3B) and LDH (Figure 3C) in the supernatant. However, pre‐treatment with koumiss extract, 2‐furoic acid, and α, α‐trehalose curbed this elevation, suggesting their efficacy in lipid breakdown enhancement. Similarly, in TAA‐induced zebrafish larvae, serum TG (Figure 6A) and TC (Figure 6B) levels were significantly higher than in controls, but pre‐treatment with these substances reduced this increase, indicating their role in lipid breakdown enhancement.

Previous studies have shown that koumiss stimulates OXPHOS by regulating mitochondrial complex I and exhibits lipid‐lowering effects (Li et al. 2021). These findings suggest that koumiss extract enhances fat absorption and improves OXPHOS sensitivity, a crucial aspect of NAFLD. Mitochondrial dysfunction, characterized by impaired fatty acid oxidation and OXPHOS, leading to oxidative stress, is a key feature of human fatty liver hepatitis. Conversely, hepatic mitochondria can adapt flexibly to metabolic conditions, preventing triglyceride accumulation and lipotoxicity in obesity. Our research focuses on how koumiss extract, 2‐furoic acid, and α, α‐trehalose promote lipid breakdown. We investigated their effects on ACAD9, ECSIT, NDUFAF1, and NDUFAF2 mRNA (Figure 4A–D), and MT‐ND6, NDUFAF1, and NDUFAF2 protein (Figure 4E,F) expression in cell models. The shift of HepARG cells to a NAFLD‐like state involves increased mitochondrial ROS production, decreased mtDNA levels, and reduced mitochondrial respiration (Bucher et al. 2018). In our experiments, compared to the control group, there was a decrease in MT‐ND6 protein expression, which partially recovered following treatment with koumiss extract, 2‐furoic acid, and α, α‐trehalose. Mitochondrial ATP production occurs through redox reactions in the ETC, coupled with phosphorylation mechanisms, known as OXPHOS. OXPHOS includes the ETC and Complex V (ATP synthase, F1F0‐ATPase) (Okoye et al. 2023).

Complex I (NADH: ubiquinone oxidoreductase or NADH dehydrogenase), the first complex in the ETC, oxidizes NADH produced by the TCA cycle, mitochondrial fatty acid β‐oxidation (FAO), and amino acid catabolism in the mitochondrial matrix (Laube et al. 2022; Vercellino and Sazanov 2022). Complex I, the largest ETC protein complex (∼980 kDa) with 45 subunits, has 37 subunits encoded by nuclear DNA (nDNA) and 7 (ND1, ND2, ND3, ND4, ND5, ND6, and ND4L) by mitochondrial DNA (mtDNA) (Yin et al. 2021; Formosa et al. 2020). NDUFAF1, ECSIT, and ACAD9 are MCIA factors, and NDUFAF2 is associated with the NAFLD pathway. In our cell experiments, compared to the control, there was regulation in ECSIT mRNA expression and effects on the expression of NDUFAF1, ACAD9, and NDUFAF2 mRNA and proteins. Post‐treatment with koumiss extract, 2‐furoic acid, and α, α‐trehalose, there was a recovery in ECSIT mRNA expression and a response in the expression of ACAD9, NDUFAF1, NDUFAF2 mRNA, and proteins. These findings indicate that koumiss extract, 2‐furoic acid, and α, α‐trehalose regulate genes related to mitochondrial complex I, participate in OXPHOS, improve lipid steatosis, and enhance energy metabolism.

Zebrafish, as an innovative animal model, offer several advantages over traditional models. They are small, cost‐effective to maintain, and have a robust reproductive capacity. In laboratory settings, zebrafish can be bred on a large scale, with a single mating yielding 100s of embryos, adequately meeting experimental needs. Their rapid development, achieving sexual maturity in approximately 3 months, allows for relatively short experimental cycles. The transparency of zebrafish embryos enables direct, live microscopic observation of organs, facilitating the study of pathological changes in vivo. Genetic research has disclosed a remarkable similarity, over 87%, between the zebrafish and human genomes, making them invaluable for large‐scale drug screening due to their ability to provide substantial sample sizes at minimal cost. Consequently, this study utilized a zebrafish model of NAFLD to mimic human NAFLD. In this zebrafish experiment, compared to the control group, the NAFLD model exhibited regulation in the expression of ECSIT, NDUFAF1, ACAD9, and NDUFAF2 mRNA. Treatment with koumiss extract, 2‐furoic acid, and α, α‐trehalose led to a noticeable recovery in the expression of NDUFAF1, ACAD9, NDUFAF2, and ECSIT mRNA (Figure 7A–D), with the impact of koumiss extract being particularly significant.

Our findings reveal that koumiss extract and its active components, including 2‐furoic acid and α, α‐trehalose, ameliorate hepatic steatosis in the HepG2 cell model by modulating the expression of MCIA proteins such as ACAD9, ECSIT, NDUFAF1, and NDUFAF2. These results provide novel insights into the molecular mechanisms underlying NAFLD and suggest that targeting OXPHOS and mitochondrial function could have significant clinical implications. By regulating the expression of key proteins like ACAD9 and ECSIT, koumiss extract and related compounds enhance OXPHOS activity within lipid metabolic pathways. This finding underscores the central role of mitochondrial dysfunction and OXPHOS in NAFLD pathogenesis.

This study has several limitations. Although the therapeutic effects of koumiss extract, 2‐furoic acid, and α, α‐trehalose on lipid metabolism and hepatic steatosis were observed in cell and zebrafish models, the clinical relevance of these findings requires further investigation. In vitro experiments may not fully replicate the complexity of in vivo environments, as individual metabolic variability could influence treatment efficacy. Additionally, the study primarily focused on mitochondrial assembly proteins and lipid metabolism without exploring other potential signaling pathways or biomarkers. Future research should encompass a broader range of biological mechanisms and validate these findings in animal models or clinical trials to enhance their translational significance and applicability.

## 5 Conclusion

In conclusion, this study provides significant preliminary evidence supporting the role of koumiss extract, 2‐furoic acid, and α, α‐trehalose in ameliorating steatosis associated with NAFLD. The findings indicate that these active components may exert beneficial effects on NAFLD by modulating the expression of MCIA‐related proteins, thereby influencing OXPHOS and lipid metabolism.

## Author Contributions

Sachula Baoyin and Qinglan Bao carried out the studies, participated in collecting data, and drafted the manuscript. Yingsong Chen and Tegexibaiyin Wang performed the statistical analysis and participated in its design. Xiaohong Bai participated in acquisition, analysis, or interpretation of data and drafted the manuscript. Xiong Ling, Biligetu Wang, and Meng Meng did literature review work. All authors read and approved the final manuscript.

## Funding

This work was supported by “Study on the key issues of curative effect of Koumiss on regional diseases of Mongolian medicine” in 2018 Supported Project of the Science and Technology program of the Department of Science and Technology of Inner Mongolia Autonomous Region, the Open Fund Project of the National‐Local Joint Engineering Research Center of Mongolian Medicine Research and Development (Grant No. MDK2023045), Natural Science Foundation of Inner Mongolia Autonomous Region (Grant No. 2024QN08066), and Science and Technology Program of the Joint Fund of Scientific Research for the Public Hospitals of Inner Mongolia Academy of Medical Sciences (Grant No. 2024GLLH1226).

## Ethics Statement

The animal experiment was executed with the agreement from the Ethics Committee of Mongolian Medicine Hospital of Xilin Gol League (YKD202201241); all methods are reported in accordance with ARRIVE guidelines.

## Consent

The authors have nothing to report.

## Conflicts of Interest

The authors declare no conflicts of interest.

## Data Availability

The authors have nothing to report.
